# Growth of ZnO Nanoparticles Using Microwave Hydrothermal Method—Search for Defect-Free Particles

**DOI:** 10.3390/nano15030230

**Published:** 2025-01-31

**Authors:** Julita Rosowska, Jarosław Kaszewski, Marcin Krajewski, Artur Małolepszy, Bartłomiej S. Witkowski, Łukasz Wachnicki, Lev-Ivan Bulyk, Piotr Sybilski, Marek Godlewski, Michał M. Godlewski

**Affiliations:** 1 Department of Physiological Sciences, Institute of Veterinary Medicine, Warsaw University of Life Sciences—SGGW, Nowoursynowska 159, 02-776 Warsaw, Poland; michal_godlewski@sggw.edu.pl; 2 Institute of Physics PAS, Polish Academy of Sciences, Al. Lotników 32/46, 02-668 Warsaw, Poland; kaszewski@ifpan.edu.pl (J.K.); bwitkow@ifpan.edu.pl (B.S.W.); lwachn@ifpan.edu.pl (Ł.W.); levivanbulyk@gmail.com (L.-I.B.); psybil@ifpan.edu.pl (P.S.); godlew@ifpan.edu.pl (M.G.); 3 Institute of Fundamental Technological Research, Polish Academy of Sciences, ul. Pawińskiego 5B, 02-106 Warsaw, Poland; mkraj@ippt.pan.pl; 4 Faculty of Chemical and Process Engineering, Warsaw University of Technology, ul. Waryńskiego 1, 00-645 Warsaw, Poland; artur.malolepszy@pw.edu.pl

**Keywords:** zinc oxide (ZnO) nanoparticles, microwave hydrothermal method, microwave-assisted synthesis, near-band-edge (NBE) emission, deep-level emission (DLE), luminescent properties of ZnO, photoluminescence (PL), cathodoluminescence (CL), defect-related luminescence

## Abstract

This study investigated the influence of chemical reagent selection on the properties of ZnO nanoparticles synthesized using the microwave-assisted hydrothermal method to control the intensities of near-band-edge (NBE) and defect-related deep-level (DLE) emissions. Two zinc precursors—zinc nitrate and zinc chloride—along with three different precipitating agents (NaOH, KOH, and NH_4_OH) were used. ZnO nanoparticles from the ZnCl_2_ precursor exhibited two orders of magnitude higher NBE/DLE intensity ratio compared to those obtained from zinc nitrate characterized by a higher contribution from defect-related emissions. Chlorine ions in ZnO nanoparticles play a key role in passivating defects by forming V_0_-Cl_2_ complexes, quenching luminescence associated with oxygen vacancies (V_0_). Thermal treatment in a nitrogen atmosphere enhanced defect-related luminescence, possibly due to chlorine atom diffusion. This study highlights a successful synthesis of ZnO nanoparticles with low defect-related luminescence (DLE) achieved via the microwave-assisted hydrothermal method, a result rarely reported in the literature. The results emphasize the importance of reagent selection in controlling the morphology and optical properties, especially the defect density of ZnO nanoparticles. Optimizing these properties is crucial for biomedical applications such as bioimaging, antibacterial treatments, and photocatalysis.

## 1. Introduction

Due to their wide range of applications, including the use in photocatalysis [1,2], optoelectronics [3,4], and gas sensing [5,6], zinc oxide (ZnO) nanoparticles are a subject of constant interest. One up-and-coming area is their potential use in biology and medicine, particularly in drug delivery systems [7,8,9], the supplementation of trace elements such as exogenous iron [10,11], antibacterial treatments [12,13], or after the additional introduction of rare-earth ions for bioimaging [14,15]. Their low toxicity, biocompatibility, and demonstrated solubility in ionic forms in physiological fluids [16,17] make them a promising solution for elimination from living organisms.

When designing materials for biomedical applications, the choice of synthesis technique is crucial. The manufacturing process should be efficient, easy to implement, and not require harmful precursors. It should also allow for the production of nanoparticles with a predetermined morphology, size, and adequate optical properties. Despite the progress in nanotechnology, meeting these requirements remains challenging.

The microwave-assisted hydrothermal technique, which belongs to the category of solution-based methods, also known as wet methods, was chosen to produce the materials discussed in this work. The earliest studies on microwave-assisted hydrothermal crystallization were conducted in the 1990s and primarily focused on synthesizing simple oxides such as TiO_2_, ZrO_2_, and Fe_2_O_3_ [18]. Although this technique for producing ZnO nanomaterials has received much attention so far [19,20,21,22,23], a unified procedure still needs to be adopted for producing nano-objects. This lack of standardization hinders process repeatability and the production of materials with strictly defined characteristics, which is essential, among other things, in the context of biomedical applications. The incomparability of the procedures of individual syntheses is related to the use of different process parameters, types of reagents, and even equipment [24]. On the other hand, hydrothermal and solvothermal methods offer the possibility of obtaining ZnO nanoparticles characterized by different crystal shapes, various morphology, and unique optical properties. In such methods, controlling these properties of the nanoparticles is possible by modifying individual synthesis parameters, such as process temperature and pressure, the chemical composition of the starting solution, reaction duration, and pH [13,25,26,27,28,29].

One of the key challenges in ZnO nanoparticle engineering is controlling their defect structure and concentration. ZnO is characterized by a wide band gap (~3.37 eV) and high exciton binding energy (~60 meV) at room temperature [29], which promotes efficient near-band-edge (NBE) emission processes. However, the luminescent properties of ZnO are highly influenced by defects in its crystal structure, to which it is reasonably susceptible. The emission spectrum of ZnO features two main bands: a near-band-edge emission (NBE) and a deep-level emission (DLE). The first one, located around ~370–380 nm (corresponding to an energy of ~3.35–3.37 eV), is attributed to free excitons (FXs), excitons bound to acceptors and donors, two-electron satellites (TES), and shallow donor–acceptor pair transitions [30]. The second, broader DLE band appears within the (2.0–2.8) eV range, corresponding to wavelengths of (620–443) nm, and is linked to deep defects in the crystal lattice.

Despite extensive research on ZnO defect luminescence, its origin remains unclear. It is currently assumed that green luminescence in ZnO can be associated with factors such as zinc vacancies V_Zn_ [31,32,33], oxygen vacancies V_o_ [34,35], zinc interstitial ions Zn_i_ [36], oxygen antisite defects O_Zn_ [37], and surface impurities [38]. Additionally, ZnO can exhibit yellow (~2.2 eV, ~564 nm) [39,40] and red (~1.8 eV, ~689 nm) [41] emissions. Yellow luminescence is associated with interstitial oxygen and frequently appears in ZnO obtained from aqueous solutions, particularly under oxygen-rich conditions, as seen in hydrothermally synthesized materials. This emission is thought to arise from interstitial oxygen O_i_ or zinc hydroxide Zn(OH)_2_ [42]. Red luminescence in ZnO nanoparticles may be due to both interstitial zinc Zn_i_ [43] and interstitial oxygen or excess oxygen [44]. It is important to note that multiple defect centers are often involved in ZnO defect luminescence due to the instability and diversity of point defect types [45].

In ZnO nanoparticle synthesis, efforts are often directed toward maximizing near-band-edge emission (NBE) while reducing defect-related emission (DLE), especially for applications requiring the precise control of optical properties. Such materials are also more accessible to excite due to fewer recombination channels. However, defects are not always undesirable, particularly in nanoparticles intended for biological applications. Numerous studies suggest defect-rich ZnO materials can benefit antibacterial applications [46,47] and photocatalysis [13].

In light of this, attempts were made to produce ZnO nanoparticles with varying degrees of defectiveness. A significant challenge in this work was to obtain ZnO nanoparticles with minimal defects linked to broad emission. To achieve this, we varied the chemical composition of the starting mixture, using two different aqueous solutions of inorganic zinc salts—zinc nitrate and zinc chloride—and three precipitating agents: NaOH, KOH, and NH_4_OH. Our focus was on characterizing the physical properties of nanoparticles obtained by the microwave-assisted hydrothermal method. Considerable emphasis was placed on explaining the potential sources of differences in the degree of defectiveness in the nanoparticles, typically expressed by various I_NBE_-to-I_DLE_ emission intensity ratio values.

## 2. Materials and Methods

### 2.1. Nanoparticles Synthesis

Six types of undoped ZnO samples were created using the microwave hydrothermal method. Two types of precursors were used, namely, zinc chloride ZnCl_2_ (99.99%, Sigma-Aldrich, St. Louis, MI, USA) and zinc nitrate Zn(NO_3_)_2_∙6H_2_O (98%, Sigma-Aldrich), and three different precipitating agents: 25% ammonia aqueous solution (NH_4_OH Carl Roth), 1 M sodium hydroxide (NaOH) solution, and 1 M potassium hydroxide (KOH) solution. The procedures for preparing samples for hydrothermal synthesis consisted of several steps, and regardless of the chemicals used, they were always the same. First, the appropriate precursors were weighed and dissolved in 200 mL of distilled water at room temperature to form a clear solution. The alkalization was performed by gradually adding a selected precipitant to an aqueous zinc salt solution until the pH reached 9.5. The resulting white precipitate was washed three times with distilled water and filtered. Then, it was placed in a Teflon reactor vessel and replenished with distilled water. The hydrothermal process was carried out in an Ertec Magnum II (Poland) reactor with 700 W microwave heating. Each reaction in the reactor was conducted for 20 min. The pressure was maintained within a range of 5.9–6.1 MPa during the process. The synthesis of the reactor was precisely controlled using the Magnum V2 software (version number 1.9.8.14). Each time, the sample system was cooled for approximately 0.5 h until it returned to ambient pressure and room temperature. The product was dried at 60 °C for 20 h and ground to a uniform powder in an agate mortar.

### 2.2. Nanoparticle Characterization

To identify the crystalline phase of the obtained nanoparticles, X-ray diffraction (XRD) tests were performed using a Panalytical X’Pert Powder Diffractometer (Westborough, MA, USA) operating with a CuKα radiation (*λ* = 1.54060Å). The data were collected in the range 20° ≤ 2θ ≤ 70° with a step of 0.05° at room temperature. ZnO nanoparticle morphology was characterized by transmission electron microscopy (JEOL—JEM 1011) operating at 80 kV accelerating voltage. A scanning electron microscope (Hitachi SU-70 model) with a high resolution of 1 nm was employed to analyze the structure, surface topography, size, and elemental composition of the samples. It was also equipped with a GATAN Mono CL3 system for cathodoluminescence measurements and an EDX characteristic radiation detector. The photoluminescence (PL) studies were conducted at room temperature using the Horiba/Jobin-Yvon Fluorolog-3 spectrofluorimeter completed with a 450 W xenon lamp, iHR550 monochromator, and Hamamatsu R928P photomultiplier.

The Fourier-transform infrared (FTIR) spectroscopy measurements were performed using a Nicolet iS10 spectrometer (Thermo Fisher Scientific, Waltham, MA, USA) in attenuated total reflectance (ATR) mode on a diamond crystal at room temperature. The spectrum was collected in the 400–4000 cm^−1^ range with a 4 cm^−1^ resolution. Thermogravimetric measurements were performed in a Mettler Toledo TGA/DSC3+ analyzer in 30 mL min^−1^ argon flow and 10 °C min^−1^ heating rate. Ten milligrams of sample was placed in a 70 µL platinum crucible, whereas the empty platinum crucible was used as a reference sample. The difference between sample and reference temperatures revealed the thermal effects of the process. The degradation/oxidation/phase transition temperature was estimated from the derivative of the TG curves.

The dependence of the luminescence spectra on excitation intensity power was collected in backscattering geometry. EKSPLA (Vilnus, Lithuania) Nd: A YAG laser with an NT342 series optical parametric oscillator was used for excitation pulses. The maximum working frequency of the laser is 20 Hz, with pulse durations of approximately three nanoseconds. Measurements of the luminescence were performed by an Acton SpectraPro SP-2500 spectrometer (Princeton Instruments, Acton, MA, USA) and collected by a multichannel detector head model C10151 provided by Hamamatsu Photonics (Hamamatsu, Shizuoka, Japan). An MCD c7557-01 controller controlled the CCD.

## 3. Results and Discussion

### 3.1. Crystal Structure

**Figure 1:**
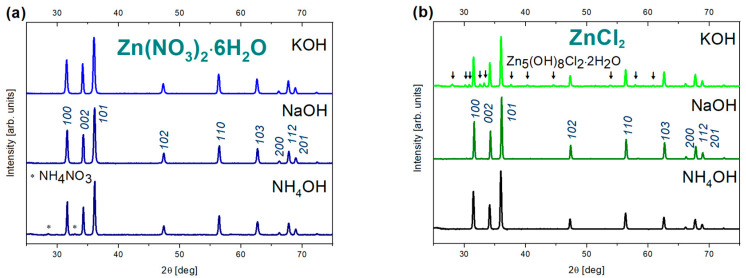
XRD patterns of ZnO samples prepared using (**a**) zinc nitrate Zn(NO_3_)_2_·6H_2_O and (**b**) zinc chloride ZnCl_2_ and precipitated with three different bases: NH_4_OH, NaOH, and KOH.

The diffraction patterns (Figure 1) indicate a hexagonal wurtzite structure typical of ZnO (PDF card no. 36-1451). However, some samples show contamination with foreign phases. In the cases of diffraction patterns for samples obtained from zinc nitrate and precipitated with NH_4_OH, there are traces of contamination related to ammonium nitrate NH_4_NO_3_ (PDF card no. 00-047-0865). The appearance of an additional ammonium nitrate phase in the XRD spectra is most likely caused by unreacted ammonium ${(NH}_{4}^{+})$ and nitrate ${(NO}_{3}^{-})$ ions. Despite filtration and repeated rinsing, some ammonium and nitrate ions may remain in the sediment, forming ammonium nitrate at a later synthesis stage. For nanoparticles synthesized from zinc chloride, the diffraction spectra show the presence of reflections from the simonkolleite phase Zn_5_(OH)_8_Cl_2_·2H_2_O (JCPDS 07-0155). This is a common impurity in ZnO nanoparticles obtained by the microwave hydrothermal method using zinc chloride for synthesis [19,48]. According to the obtained diffraction patterns, its highest content was observed for nanoparticles precipitated with KOH. The diffractograms of nanoparticles synthesized using NH_4_OH do not show the presence of zinc hydroxychloride complexes. Simonkolleite is a zinc-layered hydroxide salt in which zinc ions coordinate with hydroxide ${OH}^{-}$ and chloride ions ${Cl}^{-}$ [49]. According to XRD measurements, the type of base used has a significant impact on the formation of the simonkolleite phase. KOH and NaOH are strong bases that dissociate completely and provide many ${OH}^{-}$ ions. Their high concentration may favor the formation of simonkolleite. In turn, NH_4_OH is a much weaker base—it is only partially dissociated and provides a less significant increase in pH than KOH and NaOH.

### 3.2. Morphology Characteristics—Scanning Electron Microscopy (SEM)

**Figure 2:**
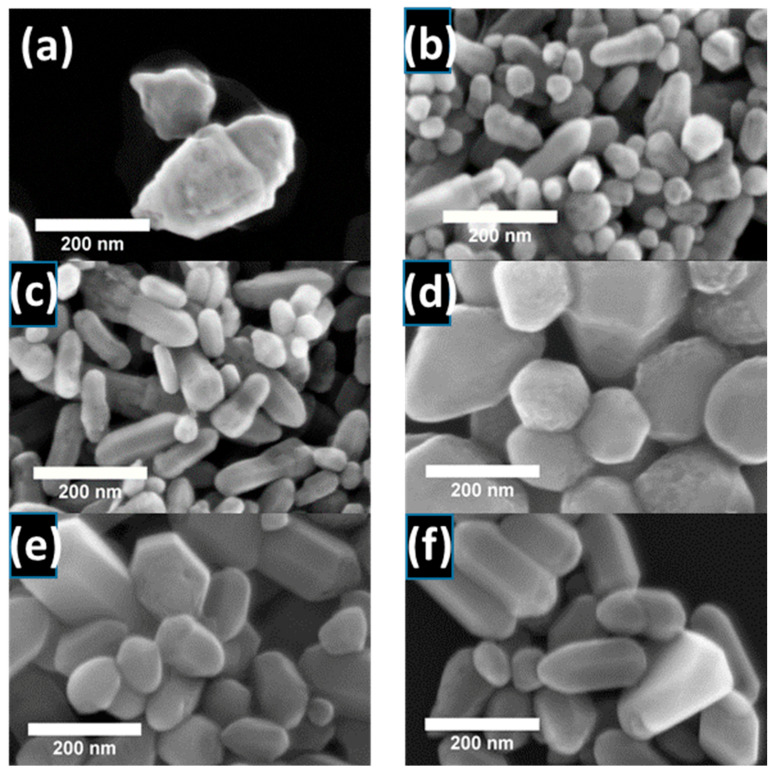
Scanning electron microscopy (SEM) images of ZnO nanoparticles obtained from Zn(NO_3_)_2_·6H_2_O precursor, precipitated with (**a**) NH_4_OH, (**c**) NaOH, and (**e**) KOH or ZnCl_2_ precursor, and alkalized with (**b**) NH_4_OH, (**d**) NaOH, and (**f**) KOH.

SEM was used to show surface structure and aggregates, and revealed that the nanoparticle morphology underwent alterations based on the chemical composition of the initial reaction mixture. The zinc precursor type (zinc nitrate and zinc chloride) and precipitating reagents (NH_4_OH, NaOH, and KOH) played a crucial role. When ZnO nanoparticles were synthesized from zinc nitrate and precipitated with NH_4_OH, irregular, surface-defective nanoparticles formed. Twin crystals fused along the c-axis (Figure 2a) appeared.

Nanoparticles derived from zinc nitrate and chloride exhibited differences in shape and size, suggesting that anion interaction with the crystal surface significantly influences growth. For ZnO nanoparticles obtained from zinc nitrate, elongated forms were observed, with shape factor values (defined as the length-to-diameter ratio, L/D) higher than those obtained from zinc chloride and alkalized with the same precipitating agent (shown later in Table 1). This is most likely because the nitrate anion is a non-coordinating ligand, meaning that it does not directly interact with the crystal surface, as it does not form strong interactions with the central ions. Growth is preferred along the c-axis (0001 plane) in the hexagonal wurtzite structure. Since nitrate anions are non-coordinating, they do not significantly modify the preferential growth direction along the c-axis [50]. In contrast, more isotropic crystal growth was observed for nanoparticles obtained from zinc chloride. This was particularly noticeable in the case of nanoparticles obtained from zinc chloride and precipitated with NaOH (Figure 2d). This is likely because the halide anion Cl adsorbs more strongly on certain crystal surfaces [50], altering their surface energy and making growth in those directions less energetically favorable. This leads to inhibited growth in specific directions. As a result, the nanoparticles adopt a more rounded shape. When using zinc chloride, the SEM images revealed objects with larger average sizes (Figure 2 and Table 1).

### 3.3. Grain Size Distributions

**Figure 3:**
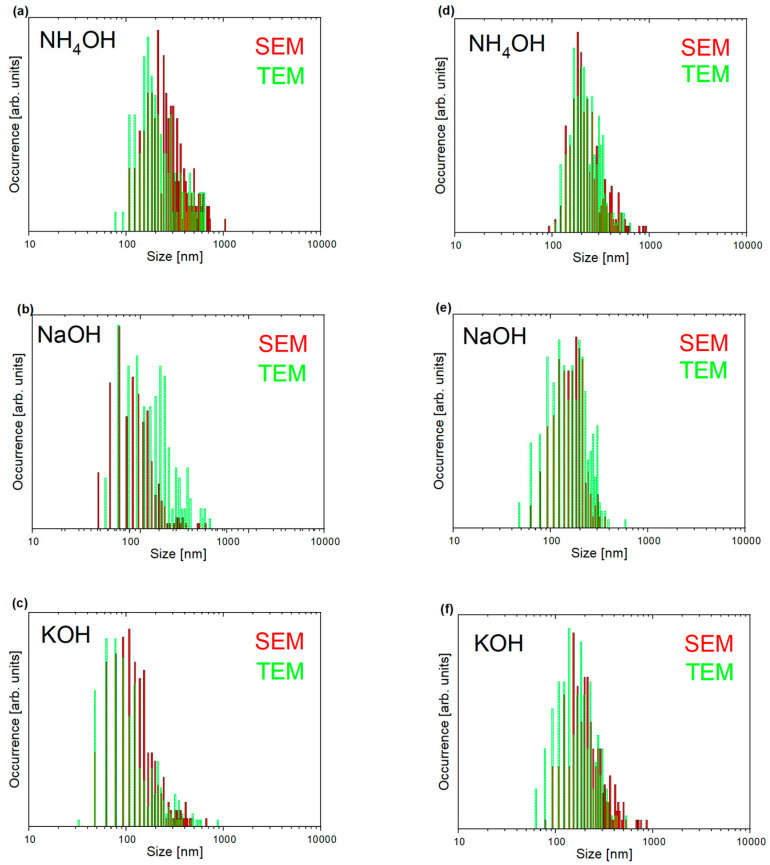
The size distributions for ZnO nanoparticles were obtained using SEM and TEM methods. Nanoparticles synthesized from Zn(NO_3_)_2_·6H_2_O precursor, precipitated with (**a**) NH_4_OH, (**b**) NaOH, and (**c**) KOH or ZnCl_2_ precursor, and alkalized with (**d**) NH_4_OH, (**e**) NaOH, and (**f**) KOH.

The figures below (Figure 3a–f) contain histograms of nanoparticle sizes obtained using various zinc salts and precipitation agents. The data come from electron microscopy images: scanning (green histograms) and transmission (red histograms; TEM images previously not shown).

Table 1 summarizes the average sizes obtained by three experimental methods: SEM, TEM, and XRD. In the case of images acquired from electron microscopy, the most extended edges of the observed objects were measured. The XRD average crystallite sizes (MCS) from XRD measurements were estimated using the approximate Scherrer method, which relates the crystal size to the diffraction peak width. Calculations were performed for the (002) reflection, corresponding to the c-axis of the ZnO crystal.

The sizes observed from SEM and TEM images are very close. The differences are relatively small for most samples (within a range of 4–49 nm). Notably, the obtained MCS sizes for most nanoparticles are smaller than those observed in SEM and TEM images, suggesting that the nanoparticles are polycrystalline in nature. The XRD method provides information about the smallest objects in the sample without considering whether the size concerns isolated objects or those grouped into larger wholes resulting from agglomeration or aggregation. The obtained data suggest that the electron microscopy images represent agglomerates or aggregates composed of several smaller primary particles rather than individual crystallites. The larger MCS size (219 nm) is only for nanoparticles prepared using zinc chloride and sodium hydroxide. This can indicate larger primary crystallites or a different growth mechanism. As previously observed in SEM images, zinc nanoparticles from the ZnCl_2_ precursor exhibit larger sizes than those prepared using zinc nitrate. This correlates with XRD tests, according to which the growth in size is also noticeable.

### 3.4. Chemical Composition and Thermogravimetry

#### 3.4.1. EDX Measurements

The elemental composition was analyzed qualitatively and quantitatively by energy-dispersive X-ray spectroscopy, with the results detailed in Table 2.

In samples derived from zinc(V) nitrate, a noticeable deficiency of zinc atoms compared to oxygen atoms (Table 2) suggests the presence of zinc vacancies (V_Zn_) or, for example, surface hydroxyl groups (Zn-OH) and their associated ions (e.g., ${Zn-OH}_{2}^{+}$ or ${Zn-O}^{-}$). Synthesis in an aqueous environment promotes the formation of surface hydroxyl groups. Additionally, nitrogen was detected within the samples, likely originating from the reagents used in the synthesis process (zinc nitrate and NH_4_OH). The highest nitrogen content was observed in samples synthesized using zinc nitrate and ammonia water, which indicates that it comes from the substrates used. Similarly, chlorine content was detected in all zinc chloride-based nanoparticles. An exceptionally high content (up to 10.7 atomic percent) is visible in the NaOH-precipitated sample. This may indicate the formation of a significant amount of separate phases like, for example, simonkolleite. On the other hand, the sample alkalized with KOH, which showed the most significant amount of simonkolleite phase according to the XRD results, at the same time, contained much less chlorine (3.2 atomic percent) according to the EDX tests. The chlorine content may be connected with separate phases and other forms like adsorbed surface chlorine species, chlorine in the form of complexes with structural defects, or amorphous phases that are not detectable by XRD studies. Point EDX analysis can only indicate the local composition, which may not represent the entire material in the case of the uneven distribution of a given element. It is important to remember that the results obtained may be subject to significant errors and only reflect general trends in the elemental composition of the samples. This is because the method used is not highly suitable for the quantitative analysis of light elements and has limited detection capabilities, making the precise quantification of these elements in the sample much more challenging.

#### 3.4.2. TGA-DTA Measurements

**Figure 4:**
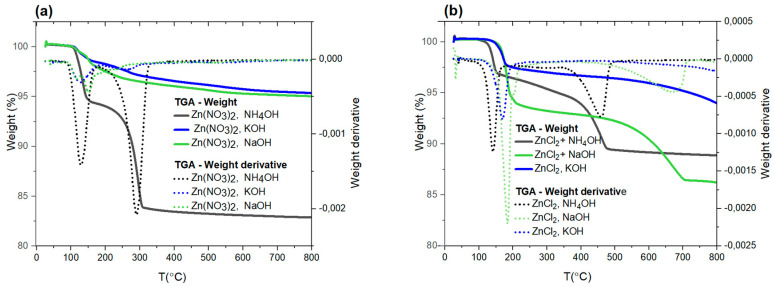
Curves obtained from thermogravimetric measurements: dependence of mass on temperature T (solid lines) and differential thermogravimetric curve (dotted lines). Results were obtained for samples made from zinc nitrate (**a**) and zinc chloride (**b**) with various precipitating agents: NH_4_OH, KOH, and NaOH.

The decrease in mass of gradually heated ZnO nanoparticles results from the release of volatile products. By raising the temperature in a controlled manner, we observe the decomposition of individual chemical compounds that make up the nanoparticles. Thermogravimetric tests are, therefore, helpful in determining the composition of the tested materials and can provide information about the chemical reactions occurring due to increasing temperature. Both samples obtained from zinc nitrate and zinc chloride were subjected to thermogravimetric analysis. The curves obtained from thermal gravimetry measurements recording the mass loss as a function of temperature are shown in Figure 4a,b. Additional differential thermogravimetric curves (Figure 4, dotted lines) are included to facilitate the interpretation of the results.

In the case of ZnO nanoparticles in which the source of Zn^2+^ ions was nitrate, and the precipitant was NH_4_OH, up to a temperature of 100 °C, there is a plateau region in which there is no change in mass due to the release of volatile sample components. The first straightforward thermal process is associated with a temperature of 128 °C, corresponding to the endothermic peak visible in the DTA (differential thermal analysis) curve (Figure 4a, black line). This is most likely related to the thermal decomposition of Zn(OH)_2_ into ZnO. According to the available literature, this process occurs in the temperature range (110–140) °C [51]. The zinc hydroxide phase (PDF card for Zn(OH)_2_: 00-048-10-66) cannot be identified in the X-ray diffraction spectra of the nitrate samples due to the overlap of most of the peaks with ZnO wurtzite.

In the same sample, in the temperature range from 150 to 300 °C, a significant weight loss occurs (nearly 10%), most likely related to the release of stoichiometric nitrogen oxides (NOX, Nitrogen OXides) from the sample. In this range, the thermal decomposition of ammonium nitrate NH_4_NO_3_ may occur, which, in the presence of metal oxides, may begin at ~145 °C. Since it is a salt of a strong acid and base, it dissolves well in water. It can, therefore, form large crystals whose crystal structure should be reflected in the form of sharp peaks in the XRD spectra. In the previously shown diffractograms of nanoparticles obtained from zinc nitrate and precipitated with NH_4_OH (Figure 1), traces of impurities appear in the low-angle range. These are peaks located around 2θ = 22°, 28°, and 32°, which, according to PDF card no. 00-047-0865, may correspond to this compound. Their presence was undetected in the remaining diffraction spectra from the discussed measurement series. It is worth noting that in the case of nanoparticles precipitated with NH_4_OH, the weight loss is the highest, resulting from the release of hydroxyl groups, stoichiometric nitrogen oxides, and ammonium groups. They are absent in the remaining samples, also synthesized with zinc nitrate, but precipitated with potassium or sodium hydroxide. The more considerable mass loss in the NH_4_OH-precipitated sample could potentially influence the defect chemistry of ZnO nanoparticles, resulting in, for example, oxygen vacancies or nitrogen incorporation into the lattice. In turn, within samples synthesized with NaOH and KOH, various complexes of zinc and OH groups with sodium, potassium, and zinc may form, such as ${Na}_{2}{Zn(OH)}_{4}$, $K_{2}Zn{\left(OH\right)}_{4}$, ${{Zn}_{2}(OH)}_{6}^{2-}$, and ${Zn(OH)}_{6}^{4-}$. The significantly smaller mass loss (~4%) observed here likely indicates surface-level changes. Furthermore, a gradual mass loss occurs from 300 °C up to the end of the measurement range (T = 800 °C). Within this range, no significant changes occur in the examined materials.

In the case of nanoparticles obtained from zinc chloride, mass loss as a function of temperature (Figure 4b) for each tested nanoparticle occurred stepwise. The highest—reaching as much as 13% of the initial mass—appears in the sample obtained from zinc chloride precipitated with sodium hydroxide NaOH (green line in Figure 4b), while the lowest—approximately 5%—is visible in the curve for nanoparticles precipitated with KOH (blue line). Nanoparticles with the highest weight loss, according to EDX research, also have the highest chlorine content (Table 2).

Due to the presence of simonkolleite Zn(OH)_8_Cl_2_·2H_2_O (Card No. 010721444) in the X-ray diffraction patterns of NaOH- and KOH-precipitated samples, a gradual transformation involving several overlapping intermediate stages can be expected as the temperature increases. The thermal conversion of this compound to ZnO is a complex process that has not yet been fully explained. According to most existing studies, this transformation proceeds by forming Zn(OH)Cl as an intermediate product, decomposing into hydrated ZnCl_2_. However, this process is complicated because both Zn(OH)Cl and ZnCl_2_ are hygroscopic compounds and may undergo hydrolysis. Nonetheless, a generalized decomposition scheme of simonkolleite leading to pure ZnO, derived from a review of relevant studies, includes the following reactions (1–4) [52]:

1.
${Zn}_{5}{\left(OH\right)}_{8}{Cl}_{2}\cdot H_{2}O\to 3ZnO+2\beta \text{-}Zn\left(OH\right)Cl+4H_{2}O\left(\sim 170\;{}^{\circ}C\right);$

2.
$2\beta \text{-}Zn\left(OH\right)Cl\to ZnO+{ZnCl}_{2}\cdot 0.25H_{2}O+0.75H_{2}O\left(\sim 220-230\;{}^{\circ}C\right);$

3.
${ZnCl}_{2}\cdot 0.25H_{2}O\to 0.25ZnO+0.5HCl+0.75Zn{Cl}_{2}\left(above\;230\;{}^{\circ}C\right);$

4. Sublimation with possible hydrolysis of $Zn{Cl}_{2}(\sim 450\;{}^{\circ}C\;and\;higher)$ [52].

The first distinct decomposition stage occurs in the temperature range of (150–210) °C for samples precipitated using NaOH and KOH. This corresponds to prominent peaks observed in the differential thermogravimetric curve at T = 170 °C (KOH sample) and T = 183 °C (NaOH nanoparticles), likely related to the initial decomposition stage of Zn(OH)_8_Cl_2_·2H_2_O, according to Reaction (1). However, it is essential to note that simonkolleite decomposition depends on experimental conditions and may proceed differently under varying gas flow parameters. Interpretation challenges also arise due to overlapping mass loss processes from various chemical compounds. The TGA results do not unambiguously identify the substances released during heating. To achieve this, the additional use of a mass spectrometer would be necessary to enable a more precise analysis of the evolved gases.

The mass loss observed at higher temperatures (max. 460 °C on the DTA curve) for nanoparticles precipitated with NH_4_OH likely corresponds to the release of ZnCl_2_. For samples prepared with NaOH, the peak associated with this phenomenon shifts to higher temperatures (670 °C). Notably, control X-ray diffraction measurements of samples annealed at 800 °C (not shown) confirmed the complete disappearance of foreign phases present in the initial material.

### 3.5. Spectroscopic Properties

#### 3.5.1. Fourier-Transform Infrared Spectroscopy (FTIR)

**Figure 5:**
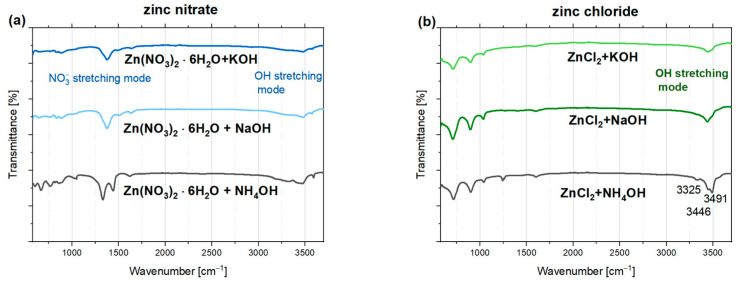
FTIR transmittance spectra of ZnO samples prepared using (**a**) zinc nitrate (V) and (**b**) zinc chloride.

ZnO has high sensitivity, and its surface can attract and be covered by water vapor, hydroxyl groups, carbon dioxide, or other chemical groups and complexes adsorbed onto the nanoparticles. These adsorbed species can significantly alter the physicochemical properties of the nanoparticles, potentially affecting their luminescence and modifying their surface. Fourier-transform infrared spectroscopy was used to obtain information about the composition of nanoparticles and to identify the functional groups existing on the surface of materials. The FTIR transmittance spectra shown in Figure 5 depend on the type of zinc precursor used during synthesis. The observed signals correspond to various functional groups present in the initial precursors. There is significantly less variation in the spectra when the same zinc precursor (chloride or nitrate) is used but with different precipitating agents—NH_4_OH, NaOH, and KOH. The infrared spectrum bands characteristic of ZnO, related to metal-oxygen (Zn-O) stretching vibrations, typically fall within 300–680 cm^−1^. Due to technical reasons, the resulting graphs only cover a small part of this measurement range, making unambiguous interpretation challenging.

The absorption band from ~3300 to approximately 3600 cm^−1^ is visible in all tested samples. It corresponds to stretching vibrations between hydroxyl groups and water molecules on the surface and between ZnO layers. Notably, this band appears at lower wavenumber values than the O-H stretching vibrations in water (3600 cm^−1^). This shift may be attributed, among other factors, to the forming of hydrogen bonds involving interlayer water and “guest” anions [53]. The presence of absorbed water in the nanoparticles may also be indicated by a weak absorption band around 1640 cm^−1^, attributed to the bending modes of H_2_O molecules or water-coordinating ${NO}_{3}^{-}$ groups. Additionally, peaks at a frequency of 3574 cm^−1^ were previously observed in the spectra of ZnO obtained by the microwave hydrothermal method. The interpretation of this stretching mode connected it to a defect associated with hydrogen consisting of a single O-H bond oriented along the c-axis of the crystal [54]. All transmittance spectra of ZnO samples derived from zinc nitrate exhibit a distinct peak around 1380 cm^−1^, corresponding to the asymmetric stretching vibrations of ${NO}_{3}^{-}$ groups. Additionally, two maxima can be identified at approximately 1328 cm^−1^ and 1442 cm^−1^ for samples precipitated with NH_4_OH. The presence of these peaks is related to the interactions between nitrate groups ${NO}_{3}^{-}$, ammonium groups $\left({NH}_{4}^{+}\right)$, and water molecules [55], and the existence of locally occurring NH_4_NO_3_ phases. The weakly defined peaks around 827 cm^−1^ indicate the presence of ${NO}_{3}^{-}$ groups and correspond to symmetric stretching vibrations.

For ZnO nanoparticles obtained using zinc chloride, distinct bands are observed at ~714 cm^−1^, ~898 cm^−1^, and ~1038 cm^−1^. These bands may suggest the simonkolleite phase (Zn_5_(OH)_8_Cl_2_ · H_2_O) [56] and correspond to the rocking vibrations of ZnOH, which are characterized by high intensity in the zinc chloride hydroxide monohydrate spectra. The positions of these lines vary depending on the base used to precipitate aqueous solution of zinc chloride. Another study attributes the similarly located bands (~720 cm^−1^ and ~889 cm^−1^) to the stretching vibration modes of chloride ions [57].

Fundamental OH stretching modes, usually observed from 3260 to 3600 cm^−1^ [56], are more intensive in nanoparticles prepared with zinc chloride. The line at ~3325 cm^−1^, observed in the spectra of nanoparticles alkalized with NH_4_OH, is often interpreted as a signal associated with hydrogen in zinc vacancies, supported by both theoretical and experimental studies [54,58,59]. The splitting of the band related to O-H stretching vibrations into two lines at 3446 cm^−1^ and 3491 cm^−1^ in the spectra of samples synthesized with NH_4_OH is likely due to interactions between hydroxyl and ammonium groups.

It is worth noting that the previously mentioned visible vibrational modes from residual chlorides and the strong hydroxyl group band may be associated with the presence of simonkolleite. This fact is further corroborated by XRD analysis, which reveals extra peaks matching Zn_5_(OH)_8_Cl_2_ · H_2_O (ICSD No. 77-2311) in the diffractograms of samples derived from zinc chloride and precipitated with KOH or NaOH (Figure 1b). These peaks are not observed for the nanoparticles precipitated with NH_4_OH, possibly because the phase content of simonkolleite in this sample is too low to be detected by XRD. Nonetheless, it is notable that the intensity of bands indicative of chloride compounds, Cl^−^ ions incorporated into the crystal structure, or surface-bound Cl^−^ residues varies between samples. The weakest bands are observed in the KOH-precipitated sample, where paradoxically, the highest simonkolleite content is seen in the diffractograms. It should be considered that nanoparticles have a high surface-to-volume ratio, and the vibrations of surface particles are more pronounced. Therefore, the surface signal will dominate. The stronger signal from residual chlorides or hydroxyl groups in nanoparticles precipitated with NaOH and NH_4_OH may result from a higher concentration of these groups on the nanoparticle surface. In contrast, in KOH-precipitated samples, a larger amount may be incorporated into the simonkolleite structure Zn_5_(OH)_8_Cl_2_ · H_2_O, limiting their presence on the surface and possibly resulting in weaker FTIR signals. Thus, differences in the intensity of specific bands on FTIR spectra may be due to the varying distribution of functional groups (whether surface-bound or incorporated into the crystal structure).

#### 3.5.2. CL and PL Measurements

**Figure 6:**
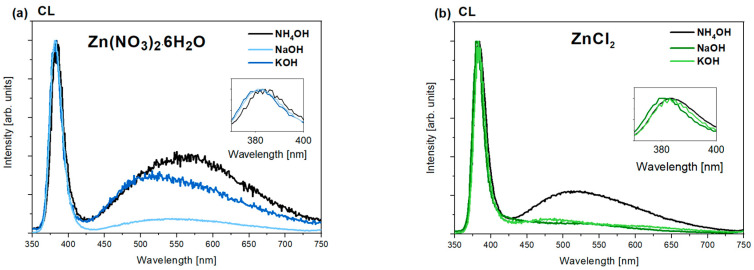
Cathodoluminescence spectra of ZnO samples prepared using (**a**) zinc nitrate (V) and (**b**) zinc chloride, and three different precipitating agents: NH_4_OH, NaOH, and KOH.

Cathodoluminescence (CL) and photoluminescence (PL) tests were performed at room temperature to examine the optical properties and degree of defectiveness of the samples. The presented cathodoluminescence spectra (Figure 6a,b) show two prominent emission bands of ZnO: NBE (~380–384 nm) and DLE (~420–720). The position of the main maximum of the DLE band for samples derived from zinc nitrate ranges from approximately 512 to 570 nm. This suggests that the defect-related luminescence of ZnO is mainly associated with the occurrence of interstitial O_i_, which is commonly found in ZnO nanomaterials synthesized in aqueous solutions under oxygen-rich conditions [60]. Excess oxygen occupies interstitial sites in such cases, forming new emission levels within the band gap. Luminescence intensity increases in the blue spectral range in the CL spectra (Figure 6) of zinc nitrate- and zinc chloride-based nanoparticles precipitated with potassium hydroxide. The blue luminescence may be connected with the transitions from interstitial zinc (Zn_i_) to the valence band [61]. The broadening of the ZnO DLE band toward the blue region of the spectrum has been previously described in the literature for ZnO doped with potassium ions synthesized via the sol–gel method [61]. In this case, the origin of the blue luminescence was associated with the presence of potassium ions in the studied materials and/or the existence of intrinsic zinc-related structural defects, such as Zn_i_ and V_Zn_ [61].

Notably, the defect band has a low contribution in the CL spectrum of nanoparticles obtained from zinc chloride and precipitated with NaOH and KOH. This suggests a high crystallographic quality of these nanoparticles. In such materials, the high intensity of the NBE band usually results from fewer carriers being trapped by defects, thereby minimizing defect-related losses and undesirable recombination processes. Consequently, more charge carriers undergo radiative recombination, contributing to NBE luminescence.

In the obtained cathodoluminescence spectra (Figure 6a,b, graph insets), a shift in the NBE (near-band-edge) emission peak is also noticeable for individual samples based on zinc chlorides and nitrates. In particular, nanoparticles precipitated with NH_4_OH show a distinct shift in the NBE emission peak towards lower energies (longer wavelengths). This is typical of samples obtained from both zinc nitrates and zinc chloride. Notably, both groups of nanoparticles precipitated with NH_4_OH exhibit the highest degree of defectivity compared to the others. The shift in the NBE maximum could be related to changes in the energy gap width due to significant defectivity or the potential doping of the semiconductor. Other possible causes include band-bending effects due to changes in particle size, the appearance of retained chemical species, and other contaminants [62].

**Figure 7:**
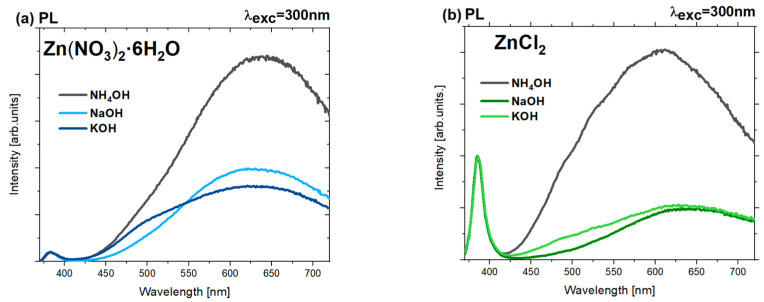
Photoluminescence spectra λ_exc_ = 300 nm of ZnO samples prepared using as a precursor (**a**) zinc nitrate and (**b**) zinc chloride.

The luminescence spectra (Figure 7a,b) obtained at room temperature with an excitation wavelength of 300 nm indicate that changing the method of sample excitation leads to a decrease in excitonic luminescence intensity and simultaneously enhances defect-related luminescence. This suggests that the relative intensities of the I_NBE_ and I_DLE_ bands strongly depend on the way and density of excitation used for the samples. In the case of cathodoluminescence, excitation is achieved through high-energy electrons, which are non-selective toward optically active excitonic or defect-origin recombination processes. It is important to note that this excitation is approximately 10^4^ times more intense than in photoluminescence (PL). For this reason, levels associated with processes with longer lifetimes (including recombination) quickly saturate, reducing the contribution from defect-related emissions. In the CL spectrum, there is thus mainly a contribution from shorter-lived processes associated with excitonic transitions, as these can efficiently occur at such high carrier densities. Therefore, the observed cathodoluminescence (CL) spectra are characterized by a more significant contribution from the NBE band (related to excitonic transitions) and a less intense defect band than the presented luminescence spectra.

However, previously observed trends are preserved. Similarly to the CL spectra, the highest contribution of defect luminescence in the PL spectrum is observed for samples synthesized from zinc nitrate. Table 3 presents the ratios of the integral intensities of near-band-edge (NBE) to defect luminescence (DLE) determined from the above luminescence spectra of all ZnO nanoparticles (Figure 7).

Notably, the data (Table 3) suggest that the choice of precipitating agent also affects the I_NBE_/I_DLE_ ratio. Among the bases used, NH_4_OH led to significantly smaller I_NBE_/I_DLE_ ratio values than NaOH and KOH, regardless of the precursors of zinc ions. The value of zinc nitrate-based nanoparticles alkalized with NH_4_OH was approximately I_NBE_/I_DLE_ ~ 0.005. In contrast, samples obtained from zinc chloride and with the same precipitant agent exhibited a value a hundred times higher, around ~0.5 (Table 3). The high concentration of defects observed in the cathodoluminescence and photoluminescence spectra is also connected with significant surface defectiveness. This is visible in the previously obtained SEM images, especially for samples synthesized using zinc nitrate and NH_4_OH (Figure 2a). Generally, the highest excitonic-to-defect emission ratio was obtained for samples synthesized using zinc chloride as a zinc source, especially for nanoparticles alkalized with NaOH (~1.59) and KOH (~1.32). These results suggest that nanoparticles with the best crystallographic quality were obtained in these cases. The above results indicate that both the type of zinc salt and the base employed in the synthesis play a crucial role in modulating the ratio between near-band and deep-level band-related emissions in the samples.

#### 3.5.3. Dependence of ZnO Luminescence Spectra on Excitation Power Density—Measurements with an OPO Laser

**Figure 8:**
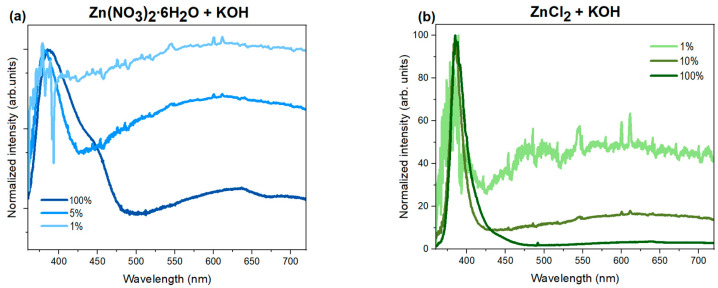
Normalized luminescence spectra of ZnO samples synthesized using zinc nitrate (**a**) and zinc chloride (**b**) precipitated with potassium hydroxide. The excitation beam power was adjusted using appropriate filters and diaphragm modulation. For samples synthesized from zinc nitrate, 1%, 5%, and 100% of the laser light was transmitted. The experiment was repeated with nanoparticles obtained from zinc chloride, where 1%, 10%, and 100% of the laser light was transmitted. In both groups of samples, a change in the intensity of the defect band is observed with the modification of the excitation beam power.

An additional experiment was conducted to verify the relationship between the relative intensities of the I_NBE_ and I_DLE_ bands and excitation density. For this purpose, nanoparticles were illuminated with a pulsed optical parametric oscillator (OPO) laser, allowing for the wavelength tuning of the output beam. The excitation power was modified using appropriate optical filters and controlled by beam modulation with a diaphragm. The following luminescence spectra (Figure 8) illustrate the results obtained with pulsed OPO laser excitation using nanoparticles obtained from zinc nitrate (Figure 8a) and zinc chloride (Figure 8b), both precipitated with potassium hydroxide.

In the above spectra excited with an OPO laser, a strong dependence on excitation power is evident, significantly affecting the intensity of individual emission bands. For both sample series, synthesized from zinc nitrate and zinc chloride, a non-linear quenching of DLE band luminescence is observed, with changes in excitation power. This supports the previous hypothesis of the saturation of recombination channels associated with defect-related luminescence. When defect states or additional levels within the band gap arise due to impurities in the crystal, the associated band intensity shows noticeable saturation with increasing excitation power. Emission from higher-energy states requires high excitation power densities to populate a more significant number of energy states. At high excitation power, as with CL measurements, the spectrum is therefore dominated by near-band-edge (NBE) luminescence, resulting from the saturation of recombination channels associated with numerous crystal defects. At the lowest excitation power densities, the band related to transitions from defect states becomes more visible. An additional effect observed for nanoparticles derived from zinc nitrate is a notable shift in the NBE luminescence peak position toward lower energies (higher wavelengths, from 379 to 393 nm) with increasing excitation power. Additionally, the shape of the NBE luminescence band changes, becoming asymmetrical, with an additional shoulder appearing on the higher wavelength side, peaking around 443 nm (2.8 eV). It can be related to potential impurity states or shallow donor levels in the ZnO nanoparticles, which are populated more at higher power densities. The broadening and shifting of the NBE band are particularly evident for samples based on zinc nitrates due to the complexity of their structure and recombination mechanisms. High excitation powers can fill shallow donor states and contribute, for example, to DAP (donor–acceptor pair) transitions. This phenomenon is practically unnoticeable for nanoparticles synthesized from zinc chloride.

#### 3.5.4. Effect of Annealing on Optical Properties and Role of Chloride Ions in Defect Passivation

**Figure 9:**
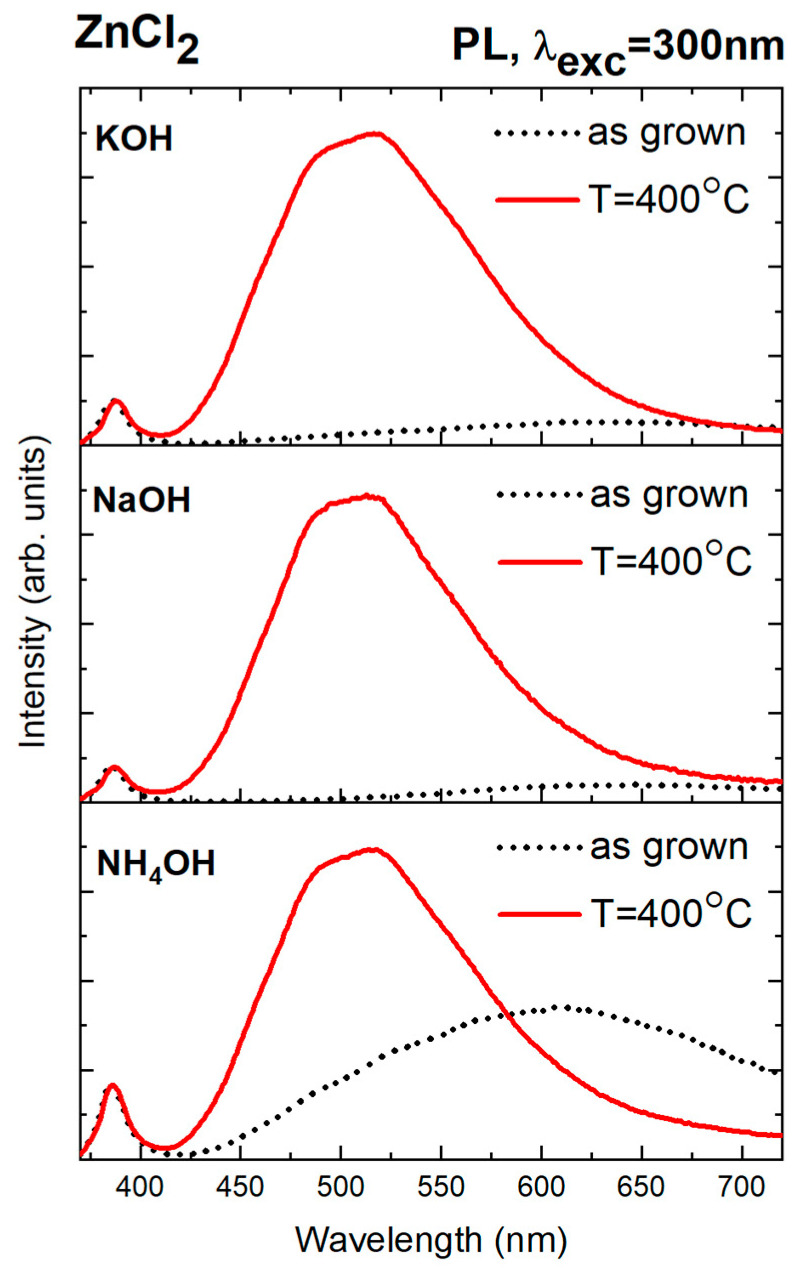
Comparison of luminescence spectra of ZnO samples synthesized from zinc chloride (ZnCl_2_) and three precipitating agents: NH_4_OH, NaOH, and KOH. PL spectra were obtained before (dotted black line) and after annealing in a nitrogen atmosphere at 400 °C (solid red line).

The luminescence and cathodoluminescence spectra shown above clearly demonstrate that it is possible to systematically control the defect states in ZnO nanoparticles by selecting appropriate chemical reagents for the reaction solution. Nanoparticles synthesized from zinc chloride and precipitated with sodium hydroxide exhibited the lowest degree of defectivity, as indicated by the highest I_NBE_/I_DLE_ ratio. These samples also contained the highest concentration of chlorine ions (according to EDX analysis, Table 2). This suggests that chlorine ions may be necessary to reduce defect-related emissions in ZnO. It is suspected that they may either fill or stabilize defects, leading to more efficient recombination and near-band-edge emission. To verify this hypothesis, nanoparticles synthesized from zinc chloride were subjected to additional thermal treatment at 400 °C in a nitrogen atmosphere. Below is a comparison of luminescence spectra obtained immediately after the hydrothermal synthesis of zinc nanoparticles from the ZnCl_2_ precursor and after additional annealing at 400 °C (Figure 9). All optical measurements were performed at room temperature.

As a result of annealing, a slight shift in the NBE band towards lower energies (longer wavelengths) was observed, from ~386 nm (~3.21 eV) to ~387 nm (~3.2 eV) for nanoparticles precipitated using NaOH and from ~386 nm (~3.21 eV) to ~388 nm (~3.20 eV) for samples alkalized with KOH. During annealing in a nitrogen-reducing atmosphere, oxygen vacancies (V_o_) and interstitial zinc (Zn_i_) exhibit a low formation energy [63]. In the obtained luminescence spectra (Figure 9), after additional thermal treatment, a significant increase in the defect band intensity was observed compared to unannealed nanoparticles, accompanied by a shift in the DLE band maximum towards shorter wavelengths (higher energies). Before heating ZnO NPs, orange-red luminescence dominates, with a maximum at ~630 nm (1.97 eV). As previously mentioned, this suggests the presence of interstitial oxygen (O_i_), typical of materials synthesized in aqueous solutions. The visible shift in the positions of the NBE and DLE bands leads to the conclusion that annealing alters the stoichiometry of the samples and modifies the mechanisms of radiative recombination processes. The heterogeneity of the defect band observed after annealing should be associated with the coexistence of defects located at different energy levels. The annealed nanoparticles are characterized by a pronounced defect band, which identifies two distinct maxima. Identifying specific defects in the green part of the spectrum remains contentious. However, according to data in the literature, the maxima positions of this band, ~496 nm (~2.5 eV) and ~520 nm (~2.38 eV), fall within the range of emissions involving oxygen vacancies (V_o_) [64,65]. The dominance of luminescence in this spectral region is likely associated with a higher concentration of V_o_ centers and concerns about emission associated with electronic transitions involving V_o_ acting as donors and holes in the valence band (D-h type). Additional thermal treatment in a nitrogen-reducing atmosphere causes a marked decrease in orange-red luminescence intensity, which suggests the diffusion of oxygen atoms that previously occupied interstitial positions.

According to the previously shown TGA results (Figure 4), heating at 400 °C causes a significant decrease in chlorine ion content in the samples, caused by its release in the gaseous phase. Simultaneously, this process is accompanied by a substantial increase in defect luminescence in the PL spectra, suggesting a correlation between chlorine content in the samples and their optical properties. Similar observations were reported in earlier studies on ZnO doped with chlorine ions [66,67]. In one of these studies, it was demonstrated that the controlled incorporation of chlorine improved the crystalline quality of ZnO. This was evidenced by the luminescence spectra of samples with the highest chlorine content, which resembled the spectra of high-quality ZnO single crystals with low defect concentration [66]. Other research [67] confirmed that doping ZnO with chlorine ions reduced defect luminescence intensity and resulted in a blue shift in the band-edge luminescence compared to undoped samples. This was attributed to the role of chlorine in passivating oxygen vacancies within the material. An oxygen vacancy (V_o_) is an anion vacancy with an effective +2 charge relative to the ZnO crystal lattice, although other charge states of V_o_ are possible. When capturing two electrons, the oxygen vacancy becomes neutral concerning the crystal lattice, or it can be singly ionized when it traps only one electron [64]. Changes in the ionization state will lead to different spectral positions for the band associated with this defect. Emission with a maximum at approximately 525 nm is attributed to complexes formed with V_o_, whereas luminescence from isolated V_o_ centers generally occurs at slightly shorter wavelengths [65]. This is explained by defect complexes involving V_o_ having energies located deeper in the band gap than isolated V_o_ centers.

In the case described, chlorine ions, present in large quantities in unannealed samples, may form complexes (Vo-Cl_2_) that can passivate possible oxygen vacancies and suppress defect luminescence from the corresponding part of the spectrum. Chlorine is a larger atom than oxygen, with a difference in ionic radii between Cl^−^ and O^2^^−^ of Δr = +38%. Chlorine can substitute oxygen in the crystal lattice or occupy interstitial positions. Specifically, chlorine ions may occupy tetrahedral and octahedral interstitial sites in ZnO with a wurtzite structure. However, based on theoretical calculations presented in previous studies, interstitial chlorine (Cl_i_) formation is energetically unfavorable in both oxygen-rich and oxygen-poor conditions [68].

The incorporation of Cl atoms depends on the growth conditions. Some studies have reported that chlorine ions can exhibit amphoteric properties—acting as an acceptor in interstitial configurations and as a donor when substituting for oxygen (Cl_O_) [69]. On the other hand, theoretical calculations have shown that both Cl_i_ and Cl_O_ levels are sufficiently deep that they likely do not directly contribute to an increase in the number of free carriers (electrons and holes) at room temperature [68].

The annealing process causes chlorine to diffuse, allowing luminescence from oxygen vacancies to become visible again in the PL spectra. An additional effect of annealing is a simultaneous decrease in oxygen ion content (Table 4) compared to the samples before annealing. This is due to the release of oxygen from the ZnO crystal lattice in the nitrogen-reducing atmosphere, which may further lead to the formation of oxygen vacancies.

The higher percentage of zinc atoms relative to oxygen atoms is most likely associated with interstitial zinc, which exhibits a low formation energy in a nitrogen-reducing atmosphere. The EDX analysis is, therefore, consistent with the previously presented optical studies and confirms the previous hypothesis.

## 4. Conclusions

This research highlights that selecting appropriate chemical reagents significantly impacts the morphology and optical properties of ZnO nanoparticles synthesized via microwave-assisted hydrothermal methods. The choice of zinc precursor (zinc nitrate vs. zinc chloride) and alkalizing agent (NH_4_OH, NaOH, and KOH) strongly affects the emission spectra and defect states. ZnO nanoparticles synthesized with ZnCl_2_ exhibited a strong NBE emission and low emission connected with defects. At the same time, zinc nitrate led to a higher contribution from the DLE band due to a more significant presence of defects, lowering the NBE/DLE intensity ratio. Chlorine ions may passivate oxygen vacancies in zinc chlorine-based ZnO nanoparticles, leading to a more pronounced near-band-edge emission. Heating such samples in a reducing nitrogen atmosphere resulted in the activation of new defects and an increase in defect band intensity in the spectral range associated with oxygen vacancies (V_O_). This phenomenon was attributed to the diffusion of chlorine atoms, which could previously form V_O_-Cl_2_ complexes, stabilizing the oxygen vacancies. Distinct excitation mechanisms, especially variations in excitation density across the applied measurement methods (PL and CL measurements), contributed to differences in the observed spectra, as evidenced by the variable contributions of the NBE and DLE bands. Therefore, it is essential to integrate multiple measurement techniques simultaneously for a reliable material evaluation, especially regarding potential application suitability. The synthesis of ZnO nanoparticles with varying degrees of defectiveness, morphology, and size may be a basis for further biological investigations focused on cytotoxicity and antibacterial properties, which will be continued in subsequent studies.

## Figures and Tables

**Table 1:** Average sizes of ZnO nanoparticles produced using various reagents and obtained by multiple measurement methods: XRD (MCS size), SEM, and TEM. The last column of the table contains the ratio of the nanoparticle length to the transverse size obtained from SEM measurements.

Precursor	Precipitation Agent	MCS (002) [nm]	Average SEM Size [nm]	Average TEM Size [nm]	Length-to-Width Ratio L/D from SEM
Zn(NO_3_)_2_·6H_2_O	NH_4_OH	107	306 ± 11	266 ± 25	1.75
Zn(NO_3_)_2_·6H_2_O	NaOH	88	130 ± 5	144 ± 6	2.4
Zn(NO_3_)_2_·6H_2_O	KOH	90	148 ± 6	144 ± 7	2.86
ZnCl_2_	NH_4_OH	107	258 ± 8	247 ± 7	1.23
ZnCl_2_	NaOH	219	169 ± 4	177 ± 5	1.88
ZnCl_2_	KOH	143	236 ± 8	187 ± 7	1.79

**Table 2:** Chemical composition of ZnO samples as measured using EDX technique.

Precursor	P. agent	Zn	O	N	Cl
Zn(NO_3_)_2_·6H_2_O	NH_4_OH	40.5 ± 0.5	54.6 ± 0.4	4.8 ± 0.4	0
	NaOH	41.5 ± 0.5	55.3 ± 0.4	3.2 ± 0.4	0
	KOH	44.4 ± 0.5	52.6 ± 0.4	2.8 ± 0.4	0
ZnCl_2_	NH_4_OH	44.2 ± 0.5	49.2 ± 0.4	2.8 ± 0.5	2.9 ± 0.1
	NaOH	40.1 ± 0.5	49.2 ± 0.4	0	10.7 ± 0.1
	KOH	50.4 ± 0.6	46.4 ± 0.4	0	3.2 ± <0.1

**Table 3:** The ratios of the intensities of near-band-edge (NBE) and defect luminescence (DLE) determined from photoluminescence spectra for the excitation wavelength λ_exc_ = 300 nm and obtained by integrating the area under the curve within specified wavelength ranges (370 to 420 nm for NBE and 420–720 nm for DLE).

Reagents Used in Synthesis		~I_NBE_/I_DLE_
Zn(NO_3_)_2_·6H_2_O	NH_4_OH	0.005
Zn(NO_3_)_2_·6H_2_O	NaOH	0.0104
Zn(NO_3_)_2_·6H_2_O	KOH	0.0128
ZnCl_2_	NH_4_OH	0.54
ZnCl_2_	NaOH	1.59
ZnCl_2_	KOH	1.32

**Table 4:** Chemical composition of ZnO samples as measured using EDX technique [atomic %] after additional annealing at 400 °C in reduction atmosphere.

Precursor	P. agent	Zn (%)	O (%)	N (%)	Cl (%)
ZnCl_2_	NH_4_OH	59.8 ± 0.6	40.1 ± 0.4	0	0
	NaOH	62.3 ± 0.6	34.7 ± 0.4	0	0
	KOH	59.7 ± 0.6	37.2 ± 0.4	0	1.3

## Data Availability

Suggested Data Availability Statements are available in section “MDPI Research Data Policies” at https://www.mdpi.com/ethics (accessed on 28 January 2025).
